# Multiple innovations underpinned branching form diversification in mosses

**DOI:** 10.1111/nph.14553

**Published:** 2017-05-04

**Authors:** Yoan Coudert, Neil E. Bell, Claude Edelin, C. Jill Harrison

**Affiliations:** ^1^ School of Biological Sciences University of Bristol Life Sciences Building 24 Tyndall Avenue Bristol BS8 1TQ UK; ^2^ Institute of Systematics, Evolution and Biodiversity CNRS Natural History Museum Paris UPMC Sorbonne University EPHE 57 rue Cuvier 75005 Paris France; ^3^ Department of Plant Sciences University of Cambridge Downing Street Cambridge CB2 3EA UK; ^4^ Royal Botanic Garden Edinburgh 20a Inverleith Row Edinburgh EH3 5LR UK; ^5^ UMR 3330 UMIFRE 21 French Institute of Pondicherry CNRS 11 Saint Louis Street Pondicherry 605001 India

**Keywords:** branching, convergent evolution, modularity, mosses, plant architecture, pleurocarpy

## Abstract

- Broad‐scale evolutionary comparisons have shown that branching forms arose by convergence in vascular plants and bryophytes, but the trajectory of branching form diversification in bryophytes is unclear. Mosses are the most species‐rich bryophyte lineage and two sub‐groups are circumscribed by alternative reproductive organ placements. In one, reproductive organs form apically, terminating growth of the primary shoot (gametophore) axis. In the other, reproductive organs develop on very short lateral branches. A switch from apical to lateral reproductive organ development is proposed to have primed branching form diversification.
- Moss gametophores have modular development and each module develops from a single apical cell. Here we define the architectures of 175 mosses by the number of module classes, branching patterns and the pattern in which similar modules repeat. Using ancestral character state reconstruction we identify two stages of architectural diversification.
- During a first stage there were sequential changes in the module repetition pattern, reproductive organ position, branching pattern and the number of module classes. During a second stage, vegetative changes occurred independently of reproductive fate.
- The results pinpoint the nature of developmental change priming branching form diversification in mosses and provide a framework for mechanistic studies of architectural diversification.

## Introduction

Morphological convergence is of major interest to evolutionary biologists, yet the mechanisms underlying convergence are poorly understood (Christin *et al*., 2010). Within the land plants, vascular plants and bryophytes have branching forms that have undergone over 420 Myr of independent evolution in distinct sporophyte and gametophyte life cycle stages, respectively (Kenrick & Crane, 1997; Edwards *et al*., 2014; Harrison, 2017a).

Sporophytic branching forms diversified from a single stemmed ancestral form with a reproductive sporangium at the tip following a gained capacity to bifurcate or bifurcate iteratively (Edwards *et al*., 2014; Harrison, 2017a), and the resultant forking architectures are thought to have minimized water loss (Niklas, 2004). More complex bifurcating architectures arose in vascular plants in response to selection for multiple traits including mechanical stability, photosynthetic efficiency, reproductive success and water‐use efficiency (Niklas, 1997, 2004). Whilst this initial stage of branching form diversification occurred during a 60 Myr period from 420 to 360 Myr ago (Ma) (Niklas, 2004; Edwards *et al*., 2014), laterally branching forms with leaves arranged around a main stem arose later in monilophytes and seed plants (Harrison, 2017a) and lateral branching is thought to optimize photosynthetic efficiency during indeterminate growth (Niklas & Kerchner, 1984).

The developmental and genetic mechanisms regulating lateral branching are well‐studied in flowering plants in which branch initiation is intimately linked to leaf initiation (Domagalska & Leyser, 2011). The site of leaf initiation reflects the position of auxin maximum formation on the dome of the shoot apical meristem (Reinhardt *et al*., 2003), and auxin transport defective mutants have disrupted leaf and branch initiation patterns (Galweiler *et al*., 1998; Reinhardt *et al*., 2003; Blakeslee *et al*., 2007; Bainbridge *et al*., 2008). Branches normally initiate as axillary meristems at the site of auxin minima that form in the crease between newly emerging leaf primordia and the meristem (Wang *et al*., 2014a,b). The outgrowth of axillary meristems as branches is regulated by environmental and endogenous cues, and long‐range auxin transport by PIN and ABCB/PGP proteins co‐ordinates an interplay between auxin, cytokinin and strigolactone in effecting branch outgrowth (Blakeslee *et al*., 2007; Domagalska & Leyser, 2011). Although the bifurcating pattern of branching in basal vascular plants is developmentally distinct from lateral branching (Bierhorst, 1977; Harrison *et al*., 2007; Harrison & Langdale, 2010; Gola & Jernstedt, 2011; Gola, 2014), long‐range polar auxin transport is a conserved property of vascular plants (Sanders & Langdale, 2013). Similarly, the genes involved in branching in flowering plants are conserved within vascular plants (Del Bem & Vincentz, 2010; Carraro *et al*., 2012; Bennett *et al*., 2014a; Lane *et al*., 2016). There is some evidence that these genes have conserved roles in regulating sporophytic branching, for instance, disruption of PIN‐mediated polar auxin transport or *TCP* gene function in moss sporophytes can induce branching (Poli *et al*., 2003; Fujita *et al*., 2008; Bennett *et al*., 2014b; Ortiz‐Ramírez *et al*., 2016; Harrison, 2017b).

The extent to which mechanisms regulating branching are shared between vascular plant sporophytes and bryophyte gametophytes, and the trajectory of gametophytic branching form diversification are unknown (Meusel, 1935; La Farge‐England, 1996; Harrison, 2017a). Mosses are the most species‐rich bryophyte lineage (Magill, 2010), their phylogenetic relationships are well‐resolved (Shaw *et al*., 2003; Buck *et al*., 2004; Bell *et al*., 2007; Quandt *et al*., 2007; Cox *et al*., 2010; Huttunen *et al*., 2013; Stech *et al*., 2013; Johnson *et al*., 2016) and their overall architecture is well sampled and documented in herbarium specimens (La Farge‐England, 1996). Furthermore, there are well‐established developmental and genetic resources for the model species *Physcomitrella patens* (Rensing *et al*., 2008) in which branches initiate by re‐specification of epidermal cells as apical cells, and branch initiation and outgrowth are continuous processes (Berthier, 1970; Coudert *et al*., 2015). Whilst a hormonal interplay between auxin, cytokinin and strigolactone has been recruited independently to regulate branch initiation patterns, auxin transport occurs via an alternative route to PINs, potentially involving plasmodesmata (Coudert *et al*., 2015). Fossil evidence dates the earliest vascular plants to *c*. 420 Ma (Kenrick & Crane, 1997), but the oldest known moss fossils date back to the lower Carboniferous (359–323 Ma; Thomas, 1972; Hübers & Kerp, 2012). Recent molecular clock estimates place the origin of mosses between 470 and 420 Ma (Clarke *et al*., 2011; Laenen *et al*., 2014), and suggest that the major clades of mosses radiated between 388–281 and 229–182 Ma (Laenen *et al*., 2014). Thus, the diversification of branching forms in vascular plant sporophytes and moss gametophytes represents a tandem experiment in evolution.

**Figure 1:**
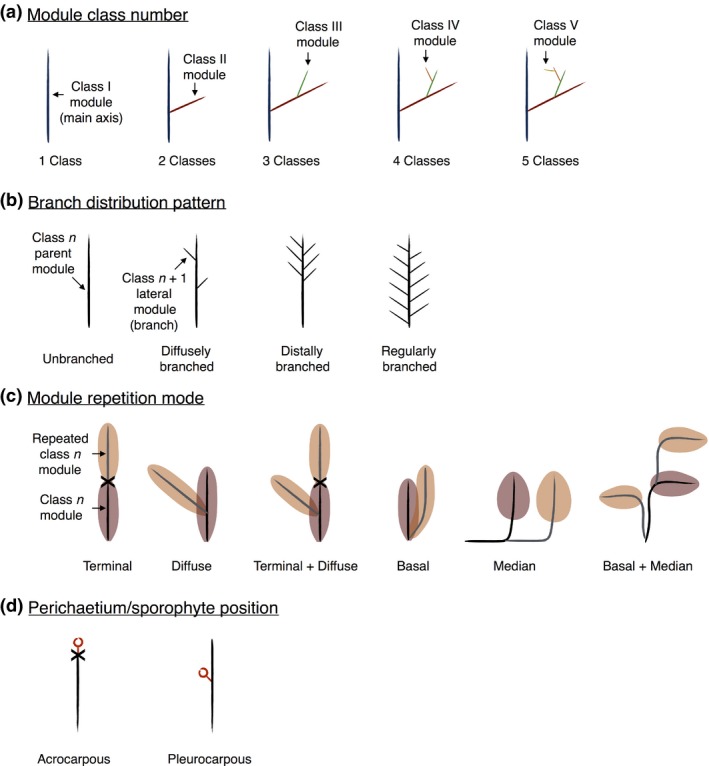
Architectural traits scored for ancestral character state reconstruction. (a) A module is defined as a portion of gametophore arising from a single apical cell (Mishler & De Luna, 1991). Modules with distinct overall morphologies were assigned to different classes, and each module class is represented with a different colour. For example, class I (primary) modules are usually wider and longer than class II (secondary) modules. (b) Branch distribution patterns characterize the topological relationship between modules belonging to distinct classes, adapted from (Barthélémy & Caraglio, 2007). For example, the main gametophore axis may be formed by a class I module (parent module) and can bear class II modules (lateral modules or branches) that are distributed along the parent module in a specific pattern. (c) Module repetition mode corresponds to the topological relationship between repeated modules belonging to the same class. The first module to develop is shown in brown, and repeat modules are shown in fawn. Crosses indicate the position of a growth arrest. A gametophore axis may comprise a single monopodial module (e.g. Supporting Information Fig. S1a) or a series of sympodial modules repeating terminally (e.g. Fig. S1o; La Farge‐England, 1996). (d) Perichaetium/sporophytes (reproductive structures) are represented by red open circles. Acrocarpy characterizes plants whose reproductive organs develop at the extremity of well‐developed, and usually primary, vegetative modules. Pleurocarpy is defined according to Newton & De Luna (1999) and see also Bell & Newton (2007): ‘All plants in which distinct vegetative leaves are lacking on fertile modules and developing archegonia are surrounded only by modified juvenile leaves, with the majority of perichaetial leaves developing after fertilisation are viewed as pleurocarps’.

There are two types of mosses defined by the position of their reproductive organs. Whilst acrocarpous mosses have gametophytic shoot (gametophore) axes that terminate in reproductive organ formation, pleurocarpous mosses have short lateral reproductive shoots with a specialized morphology (Fig. 1d; Bell & Newton, 2007). Acrocarpy is plesiomorphic within mosses and pleurocarpy is hypothesized to have been a key innovation priming branching form diversification (Bell & Newton, 2007; Newton, 2007). However, this hypothesis has not been tested in a broad phylogenetic context and the evolutionary history of moss architectural diversification remains largely unresolved.

## Materials and Methods

### Taxon sampling

One hundred and seventy‐five bryopsid mosses were selected for architectural and phylogenetic analysis, with sampling strongly skewed towards the subclass Bryidae (154 exemplars) and the superorder Hypnanae (101 exemplars). Complex branching architectures in mosses are largely restricted to the Bryidae and are most diverse in the Hypnanae (the core pleurocarps *sensu* Bell & Newton, 2007), although some also occur in the subclass Dicranidae (the Haplolepidous mosses) from which 18 exemplars were selected. Whilst other branching forms characterize distantly related groups such as *Sphagnum* and isolated members of the Polytrichopsida, these are not directly homologous to those found in the Bryopsida and were regarded as being outside the scope of this study. Within the pleurocarpous mosses, sampling was biased to maximize phylogenetic and architectural diversity by the inclusion of early‐diverging lineages at the expense of closely related and morphologically similar taxa in the highly speciose order Hypnales.

### Architectural analysis

**Figure 2:**
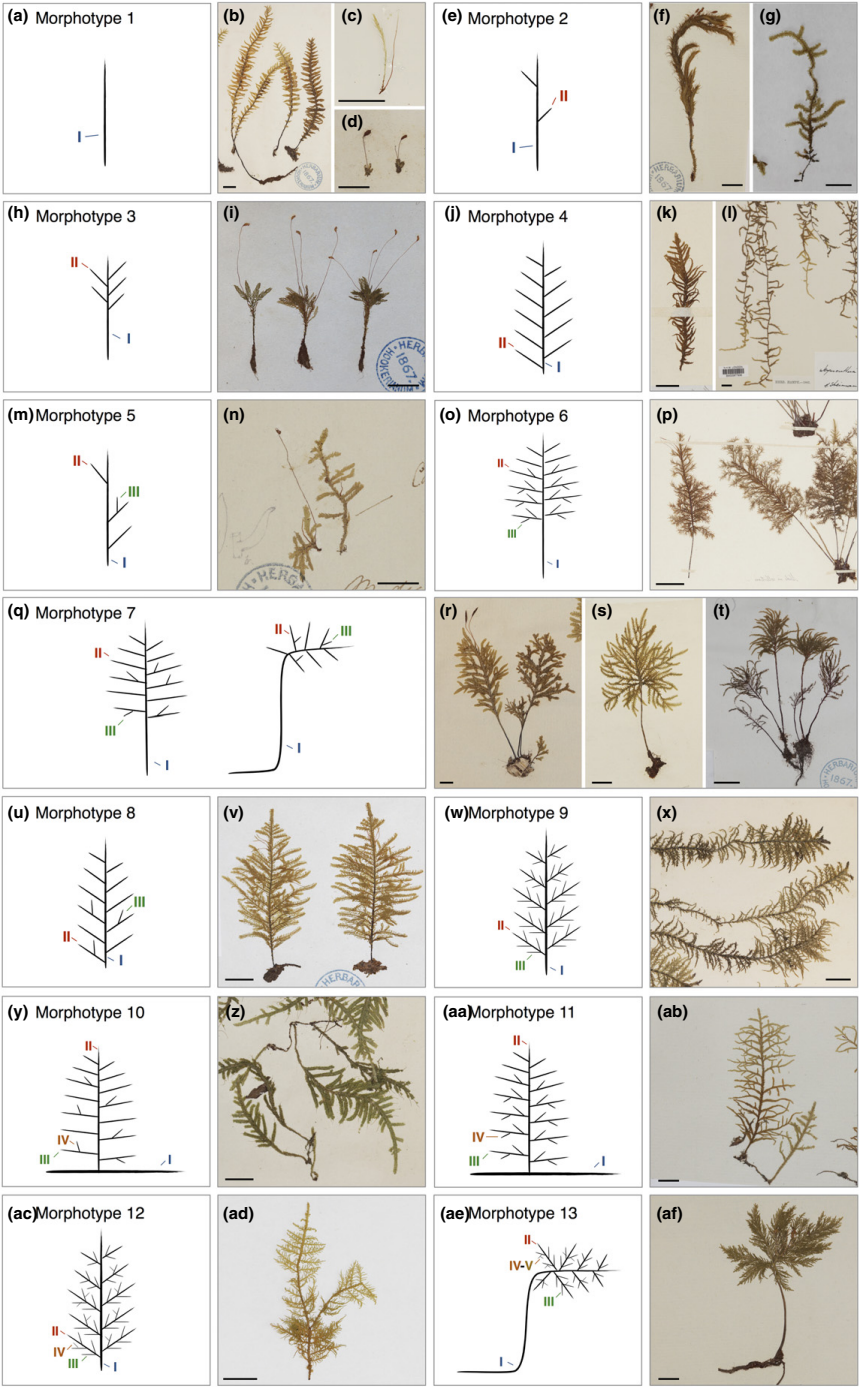
Thirteen architectural morphotypes capture most of the variation in branching form in mosses. (a–d) Species assigned to morphotype 1 (a) have one vegetative module class corresponding to the main gametophore axis. Photomacrographs of (b) *Cyathophorum bulbosum*, (c) *Hymenodontopsis stresemannii* and (d) *Funaria hygrometrica*. (e–g) Species assigned to morphotype 2 (e) have two module classes and diffuse branching patterns. Photomacrographs of (f) *Cyrtopus setosus* and (g) *Meteoriopsis reclinata*. (h, i) Species assigned to morphotype 3 (h) have two module classes and distal branching patterns. (i) Photomacrograph of *Hymenodontopsis bifaria*. (j–l) Species assigned to morphotype 4 (j) have two module classes and regular branching patterns. Photomacrographs of (k) *Abietinella abietina* and (l) *Weymouthia mollii*. (m, n) Species assigned to morphotype 5 (m) have three module classes and diffuse branching patterns. (n) Photomacrograph of *Hypnella pallescens*. (o, p) Species assigned to morphotype 6 (o) have three module classes, distal branching on class I modules and regular branching on class II modules. (p) Photomacrograph of *Pterobryella speciosissima*. (q–t) Species assigned to morphotype 7 (q) have three module classes, distal branching on class I modules and diffuse branching on class II modules. Photomacrographs of (r) *Braithwaitea sulcata*, (s) *Arbusculohypopterygium arbusculum* and (t) *Leucolepis acanthoneura*. (u, v) Species assigned to morphotype 8 (u) have three module classes with regular branching on class I modules and diffuse branching on class II modules. (v) Photomacrograph of *Lopidium concinnum*. (w, x) Species assigned to morphotype 9 (w) have three module classes and regular branching patterns. (x) Photomacrograph of *Hylocomium splendens*. (y, z) Species assigned to morphotype 10 (y) have four module classes with diffuse branching on class I and III modules and regular branching on class II modules. (z) Photomacrograph of *Orthostichella versicolor*. (aa, ab) Species assigned to morphotype 11 (aa) have four module classes, with diffuse branching on class I modules and regular branching on class II and III modules. (ab) Photomacrograph of *Pilotrichum bippinatum*. (ac, ad) Species assigned to morphotype 12 (ac) have four module classes, with regular branching on class I and II modules and diffuse branching on class III modules. (ad) Photomacrograph of *Thuidium delicatulum*. (ae, af) Species assigned to morphotype 13 (ae) have five module classes with distal branching on class I modules, regular branching on class II and III modules and diffuse branching on class IV modules. (af) Photomacrograph of *Dendrohypopterygium filiculiforme*. Numbers (I–V) indicate module classes. Bars, 1 cm.

**Figure 3:**
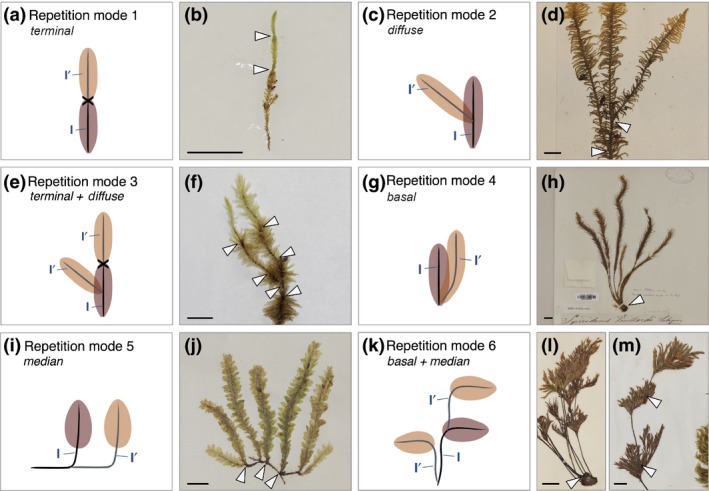
Module repetition defines a higher level of architectural organization. (a, c, e, g, i, k) Diagrams represent observed primary module repetition modes: (a) terminal, (c) diffuse, (e) a combination of terminal and diffuse, (g) basal, (i) median, or (k) a combination of basal and median. The first module to develop is shown in brown (I), and repeat modules are shown in fawn (I′). Crosses indicate the position of a growth arrest. (b, d, f, h, j, l, m) Photomacrographs of (b) *Eustichia longirostris* showing terminal repetition, (d) *Ptilium crista castrensis* showing diffuse repetition, (f) *Pulchrinodus inflatus* showing a combination of terminal and diffuse repetition, (h) *Spiridens vieillardii* showing basal repetition, (j) *Schimperobryum splendidissimum* showing median repetition and (l, m) *Pterobryella praenitens* showing a combination of basal and median repetition. White arrowheads indicate origins of primary module repetition. Bars: (b) 0.5 cm; (d, f, h, j, l, m) 1 cm.

Herbarium specimens whose identification was confirmed by the authors or other experts in the field were sampled, rehydrated when possible, dissected if necessary and drawn by hand to score architectural characters (see Figs 1, 2, 3, and Supporting Information Fig. S1) and the character matrix was verified with data from the literature when possible.

### Phylogenetic analyses

Partial sequences of the chloroplast *rps4* and *rbcL* genes and the mitochondrial *nad5* gene (including the moss and liverwort specific group I intron (Beckert *et al*., 1999)) were obtained from NCBI GenBank (http://www.ncbi.nlm.nih.gov/genbank/; Table S1) and aligned manually using phyde v.0.997 (Müller *et al*., 2011). The *nad5* gene sequences were restricted to the intron and a short section (*c*. 250 bp) of the flanking 5′ exon. Alignment was entirely unambiguous for protein coding regions and almost so for the *nad5* intron, which is relatively conserved. Sequences of *rps4* were obtained for all 175 species, whereas *nad5* was obtained for 170 of these and *rbcL* for 99.

Phylogenetic analyses were conducted using maximum likelihood (ML) and Bayesian approaches, in each case using a heterogenous generalized time‐reversible model of nucleotide substitution with gamma‐distributed rate variation between sites (GTR + G). Partitions corresponded to gene regions (*rps4*,* rbcL* and *nad5*) and were unlinked to allow parameters other than topology to vary independently. ML analysis was conducted using raxml v.7.4.2. (Stamatakis, 2006) through the raxmlgui v.1.3 front end (Silvestro & Michalak, 2012). The ‘ML + thorough bootstrap’ option within the raxmlgui (raxml option ‘‐b’ followed by an ML search) was used on the partitioned dataset, with 10 runs and 500 replicates. Bayesian analysis was conducted using mrbayes v.3.1.2 x64 (Ronquist & Huelsenbeck, 2003). In each analysis, three independent runs using the default prior settings, each with five chains (‘temp’ parameter = 0.08), were run simultaneously for 1 × 10^7^ generations with trees sampled every 1000 generations. Adequate sampling from the cold chain at stationarity and convergence of independent runs was assessed by checking that the average standard deviation of split frequencies was < 0.01, potential scale reduction factor (PSRF) values were near 1.00, effective sample sizes for each parameter were meaningful, and sampling from the posterior probability (PP) distribution was accurate as assessed by examination of log‐likelihood trace files in tracer v.1.6.0 (Rambaut *et al*., 2014). The first 50% of trees (including those from the burn‐in phase) were discarded and a majority‐rule consensus tree constructed using the remaining three samples of 5000 trees.

### Ancestral character state reconstruction

**Figure 4:**
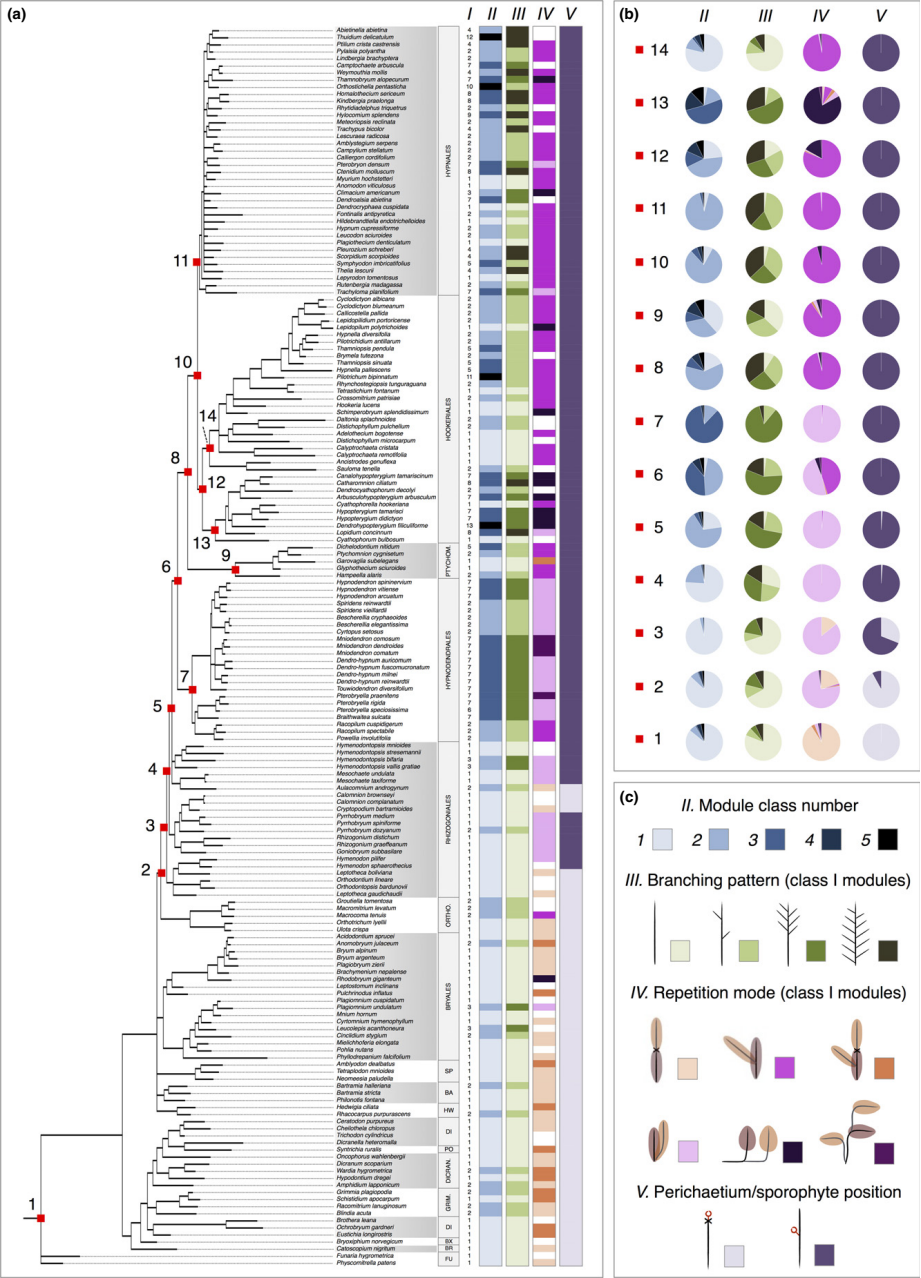
Evolutionary history of developmental innovations underpinning branching form diversification in bryopsid mosses. (a) Phylogenetic distribution of (I) architectural morphotypes, (II) number of module classes, (III) branching patterns for class I modules, (IV) repetition modes for class I modules, and (V) perichaetium/sporophyte position in 175 selected species of Bryopsida. Missing data is shown in white. Abbreviations: FU, Funariales; BR, Bryales; BX, Bryoxiphiales; DI or DICRAN, Dicranales; GRIM, Grimmiales; PO, Pottiales; HW, Hedwigiales; BA, Bartramiales; SP, Splachnales; ORTHO, Orthotricales; PTYCHOM, Ptychomniales. *Classification information*: Rhizogoniales comprise a grade of three familial clades, the Orthodontiaceae (incl. *Orthodontium lineare*), Rhizogoniaceae (incl. *Rhizogonium distichum*) and Aulacomniaceae (incl. *Aulacomnium androgynum*). Hypnanae (node 6) is a superorder comprising four orders: Hypnodendrales, Ptychomniales, Hookeriales and Hypnales. Node 3 corresponds to all Rhizogoniales and Hypnanae, node 4 corresponds to the Rhizogoniaceae, Aulacomniaceae and Hypnanae, and node 5 corresponds to the Aulacomniaceae and Hypnanae. Hookeriales (node 12) comprise the ‘core Hookeriales’ (node 14) and the family Hypopterygiaceae (node 13). (b) Bayesian reconstruction of ancestral states for characters II, III, IV and V at selected nodes. Pie charts represent mean percentage probabilities of ancestral character states from three independent analyses (Table S2). (c) Key to character states. The number of module classes ranges from 1 to 5 and primary (class I) modules may be unbranched or have diffuse, distal or regular branching patterns. Module repetition modes vary as identified in Fig. 2. Perichaetium/sporophyte position may be acrocarpous (terminal) or pleurocarpous (on short lateral branches). Note: the inference that a change in module repetition mode preceded the origin of pleurocarpy is supported by further character state reconstructions at nodes X, Y and Z that fall between nodes 1 and 2. The results from these analyses are shown in Table S2.

In order to reconstruct ancestral states of characters representing branching form diversification at key nodes, we used the ‘MultiState’ option within the program bayestraits v.2.0 (Meade & Pagel, 2014). bayestraits analyses reconstruct probabilities for ancestral states at specified nodes, using a model in which values of parameters for transition rates between states are estimated in the context of a known tree with given branch lengths. A sample of 5000 trees from the post‐burn‐in phase of one of the three mrbayes runs was used as an input for all reconstructions. For each of 14 ancestral nodes four characters were reconstructed, with five, four, six and two states, respectively (see Fig. 4 and Table S2). To obtain posterior distributions of models of evolution for each character, we used the reversible jump MCMC option within bayestraits multistate (Meade & Pagel, 2014). An exponential prior was seeded from a uniform hyperprior on the interval 0–100 (Meade & Pagel, 2014). The ‘addmrca’ command was used to generate posterior distributions of likelihoods for ancestral character states for MRCAs of selected groups of taxa. This allows posterior distributions of MRCA states to be calculated even where MRCA nodes for a group are different between different sets of trees due to phylogenetic uncertainty. Analyses were executed sequentially using command‐line batch files, each with 5 × 10^6^ iterations, a burn‐in of 1 × 10^6^ and a sample frequency of 300. Three independent runs were conducted for each reconstruction at each node. Posterior probabilities of states were calculated from posterior distributions in output files and averaged across the three runs in each case (Table S2).

## Results

### Thirteen architectural morphotypes capture most of the variation in branching form

In order to analyse branching forms in mosses and test the hypothesis introduced above, we rehydrated, photographed and drew by hand herbarium specimens of 175 species from 15 orders and 60 families of Bryopsida, the largest class of mosses (Table S1). The sampling strategy was biased towards pleurocarpous species and their close relatives to reveal branching homologies associated with the transition to pleurocarpy. *Physcomitrella patens* and *Funaria hygrometrica* were selected as distant outgroups based on their use as model taxa (Bell & Newton, 2007; Rensing *et al*., 2008). Morphotypes summarizing the architectural features of each species were prepared, allowing us to identify characteristics that were shared between species (Figs 1, 2, S1; Table S3). A module is defined as a portion of gametophore arising from a single apical cell, and modules with distinct overall morphologies were assigned to different classes (Figs 1a, S1; La Farge‐England, 1996). Typically, class I modules form the main gametophore axis, class II modules branch out from class I modules, class III modules branch out from class II modules, class IV modules branch out from class III modules and class V modules branch out from class IV modules (Fig. 1a). All forms had modular construction with up to five module classes, but most species had one (43%), two (35%) or three (20%) module classes (Fig. 1a).

The distribution of lateral modules (or branches) on parent modules (e.g. distribution of class II modules on class I modules) varied, and four main branch distribution patterns were identified (Figs 1b, S1). Parent modules were either (1) unbranched with no lateral modules, (2) diffusely branched with sparse, irregularly distributed lateral modules, (3) distally branched with modules aggregated towards the apex of the parent module or (4) regularly branched with lateral modules evenly spaced along the parent module (Figs 1b, S1). By combining the number of module classes with branching patterns we were able to define 13 architectural morphotypes that captured most of the variation in branching form between species (Fig. 2).

These were:

- Morphotype 1: species with a single unbranched module class (Fig. 2a–d).
- Morphotype 2: species with two module classes and diffuse branch patterning (Fig. 2e–g).
- Morphotype 3: species with two module classes and distal branch patterning (Fig. 2h,i).
- Morphotype 4: species with two module classes and regular branch patterning (Fig. 2j–l).
- Morphotype 5: species with three module classes and diffuse branch patterning (Fig. 2m,n).
- Morphotype 6: species with three module classes, distal branch patterning on class I modules and regular branch patterning on class II modules (Fig. 2o,p).
- Morphotype 7: species with three module classes, distal branch patterning on class I modules and diffuse branch patterning on class II modules (Fig. 2q–t).
- Morphotype 8: species with three module classes, regular branch patterning on class I modules and diffuse branch patterning on class II modules (Fig. 2u,v).
- Morphotype 9: species with three module classes and regular branch patterning (Fig. 2w,x).
- Morphotype 10: species with four module classes and a combination of diffuse branch patterning on class I modules, regular branch patterning on class II modules and diffuse branch patterning on class III modules (Fig. 2y,z).
- Morphotype 11: species with four module classes and a combination of diffuse branch patterning on class I modules and regular branch patterning on class II–III modules (Fig. 2aa,ab).
- Morphotype 12: species with four module classes and a combination of regular branch patterning on class I–II modules and diffuse branch patterning on class III modules (Fig. 2ac,ad).
- Morphotype 13: species with five module classes and a combination of distal branch patterning on class I modules, regular branch patterning on class II–III modules and diffuse branch patterning class IV modules (Fig. 2ae,af).

The architectural traits above were recorded from herbarium specimens, but plant development is environmentally responsive, and this can impact on form (Barthélémy & Caraglio, 2007). For instance, in controlled laboratory conditions *Physcomitrella patens* has a diffuse branching pattern conforming to morphotype 2 (Coudert *et al*., 2015) but *Physcomitrella patens* herbarium specimens were unbranched. We therefore consider the architectural morphotypes illustrated in Fig. 2 as landmarks in a morphospace within which species occupy bounded and potentially overlapping domains (Langlade *et al*., 2005).

### Six module repetition modes define higher levels of architectural organization

Whilst the architectural morphotypes above accounted for much variation in moss form, further variation was introduced by the repetition of modules belonging to the same class during gametophore development, thereby generating a higher level of organization (Fig. 1c). For instance, a chain of repeated modules could form by meristem arrest and subsequent initiation of a new apical cell (Figs 1, 3a,b). Such forms typically arise following gametangium formation and can be distinguished from continuously growing forms by a scar marking the point of meristem arrest between two successive modules and heteroblastic leaf series in each repeated module (Mishler & De Luna, 1991; Barker & Ashton, 2013). Using these markers, six module repetition modes were characterized (Figs 1c, 3). Module repetition was either:

- terminal as described above (Fig. 3a,b),
- diffuse, with modules developing at different positions anywhere along the initial module (Fig. 3c,d),
- a combination of terminal and diffuse (Fig. 3e,f),
- basal, with repetition occurring at the base of the initial module (Fig. 3g,h),
- median, with repetition occurring in the middle portion of the initial module (Fig. 3i,j) or
- a combination of basal and median (Fig. 3k,m).

Repetition was mainly restricted to primary (class I) module classes (Fig. S1), although secondary (class II) modules also can repeat (see Fig. S1), and many cases of diffuse repetition seemed to result from the accidental death of the primary module meristem, so were not scored. The ability to repeat primary modules at various positions offers potential to generate increasingly complex forms. Therefore, vegetative module class number, branch distribution patterns and module repetition mode contribute to the overall branching form of moss gametophores.

### Stepwise changes primed branching form diversification in mosses

In mosses, reproductive organs are either located at the apex of primary modules (acrocarpous mosses) or at the tips of highly reduced lateral modules (pleurocarpous mosses) (Fig. 1d; Bell & Newton, 2007). It is largely assumed that the innovation of pleurocarpy underpinned the radiation of branching forms in mosses (La Farge‐England, 1996; Newton, 2007), but pleurocarpy and branching innovations are often conflated (Mishler & De Luna, 1991). To dissect the contributions of pleurocarpy and vegetative branching innovations to moss diversification, we undertook ancestral character state reconstruction using the architectural traits identified in Figs 1, 2, 3. We first generated a molecular phylogeny of study species using publicly available aligned genomic sequences of *nad5*,* rbcL* and *rps4* loci (Table S1). We reconstructed phylogeny using ML and Bayesian methods, and both methods converged on similar tree topologies that were generally consistent with results published elsewhere (Buck *et al.,* 2004; Bell *et al.,* 2007; Cox *et al.,* 2010; Figs S2, S3; Notes S1). We mapped architectural morphotypes (character I), number of module classes (character II), branch distribution patterns (character III), module repetition mode (character IV) and reproductive organ position (character V) onto a consensus phylogenetic tree (Fig. 4a). To infer the sequence of architectural innovation we reconstructed ancestral character states using a Bayesian approach (Figs 4b, S4; Table S2).

Our results were consistent with previous analyses (Bell & Newton, 2007) suggesting that acrocarpy is plesiomorphic within the Bryopsida (node 1: 100% probability), and for the first time supported a single origin for pleurocarpy in a common ancestor of the Rhizogoniales and Hypnanae (node 3: 69% probability). Module repetition mode changed before the transition to pleurocarpy, and the most likely scenario was a shift from terminal to basal repetition (node 2: 75% probability) (Table S2). Architectural morphotypes were conserved between acrocarpous mosses and basal pleurocarps (Fig. S5), suggesting that the number of module classes and branch distribution patterns changed independently of pleurocarpy (nodes 1–5). Distinct architectures with two module classes and distal branching patterns originated at the divergence point between the crown Hypnanae and their sister group, the Aulacomniaceae (node 5). The data above suggest that a basal module repetition mechanism evolved before pleurocarpy, and was then co‐opted into reproductive development. Together with increasingly complex modularity and branch patterning, these innovations drove a first stage of diversification in branching form.

### Branching forms diversified further after the origin of pleurocarpy, following lineage‐specific evolutionary trajectories

**Figure 5:**
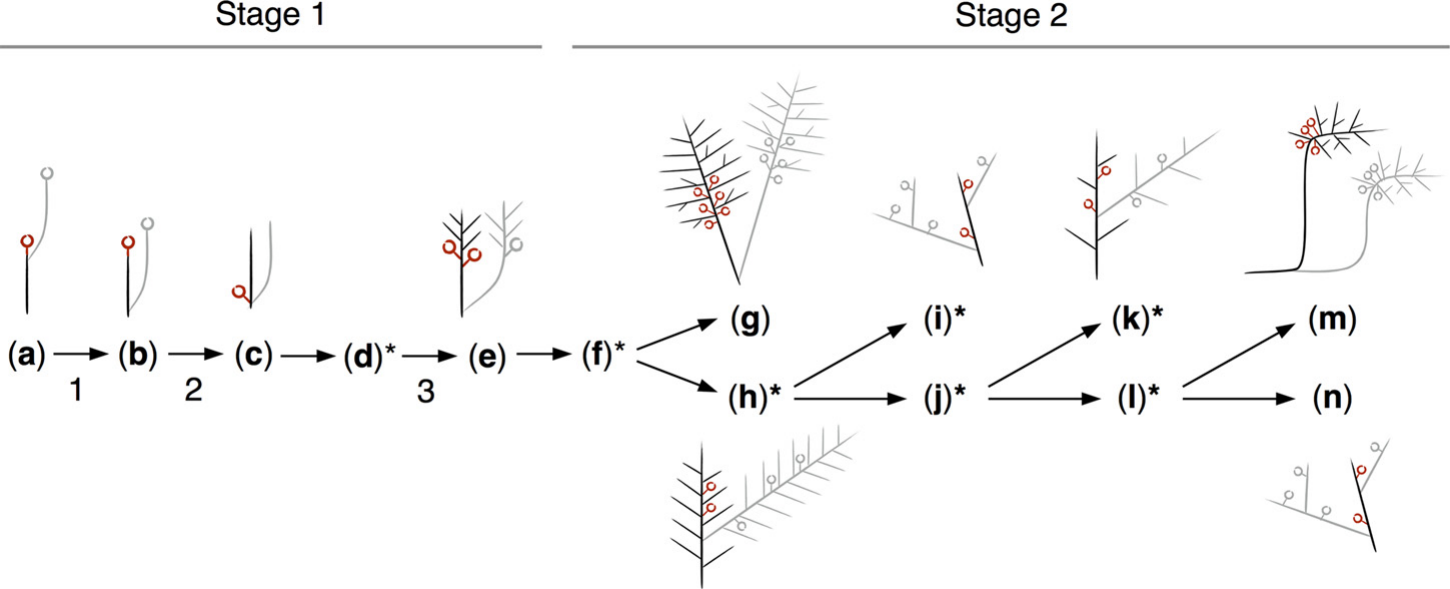
Inferred steps in architectural diversification. Diagrams represent the most likely ancestral branching forms reconstructed at nodes (a) 1, (b) 2, (c) 3, (e) 5, (g) 7, (h) 8, (i) 9, (k) 11, (m) 13 and (n) 14 of the phylogenetic tree (Fig. 4). Ancestral branching forms at nodes (d) 4, (f) 6, (j) 10, (l) 12 were not represented due to uncertainty in reconstructions (indicated by asterisks). Reproductive structures are indicated by red open circles and repeat modules are shown in grey. During a first stage of diversification these hypothetical forms evolved through stepwise changes corresponding to (1) a shift from terminal to basal module repetition, (2) a shift from acrocarpy to pleurocarpy, and (3) an increase in module class number and distal branch patterning. Uncoupled changes in the number of module classes, the distribution of lateral modules and primary module repetition modes occurred during a second stage of diversification, with frequent reversals to simpler forms.

Whilst pleurocarpy remained fixed after the origin of the Hypnanae (node 6), further aspects of branching form identified in Figs 2 and 3 were homoplastic (Fig. 4a,b). For instance, the number of module classes increased from two to three in ancestors of the Hypnodendrales (node 7: 84% probability) and Hypopterygiaceae (node 13: 51% probability), and reverted to one in the ancestor of the ‘core’ Hookeriales (node 14: 78% probability). Branching patterns showed similar homoplasy, reflected in uncertainty in ancestral state reconstruction (nodes 8–13). Diffuse, distal and regular branching patterns were frequent in crown Hypnanae, suggesting that there is no typical pleurocarpous branching habit. A basal pattern of module repetition was the most probable ancestral state in the Hypnanae (node 6: 48% probability) and Hypnodendrales (node 7: 98% probability), and shifts to diffuse (node 8: 96% probability) and median (node 13: 81% probability) module repetition modes occurred in more recently derived lineages. Thus, within the Hypnanae branching forms diversified by distinct evolutionary trajectories (Figs 4, 5, S4, S5).

## Discussion

### Two main stages in the evolutionary history of moss branching forms

The data presented here suggest that a switch in reproductive organ placement was not the sole innovation for branching form diversification in mosses. Rather, our analysis suggests that unbranched forms with terminal module repetition are likely to be ancestral within bryopsid mosses (Figs 5a, S4). Stepwise innovations in the Rhizogoniales primed a first phase of diversification in form (Bell & Newton, 2007) comprising (1) a shift from terminal to basal module repetition mode (Fig. 5b), (2) pleurocarpy – the displacement of reproductive organs from terminal to lateral positions relative to the main gametophore axis (Fig. 5c), and (3) a capacity to form multiple module classes with distal distributions (Fig. 5e). During a second phase of diversification, pleurocarpous mosses radiated by lineage‐specific and uncoupled changes in module class number, branching pattern and repetition mode, leading to the evolution of similar architectures by convergence (Figs 4, 5g–n, S4).

### Different selective pressures on branching in mosses and vascular plants

The distinct evolutionary trajectories of branching form diversification in moss gametophytes and vascular plant sporophytes are reflected in distinct developmental innovations and selective pressures in each lineage. In mosses, branch initiation reflects specification of a new apical cell on the moss gametophore axis rather than apical dichotomy (Coudert *et al*., 2015), and the innovation of basal repetition (Fig. 5b) and the formation of multiple module classes (Fig. 5e–n) reflect shifts in the position of respecified cells. Some multi‐modular moss forms observed produce planar leaf‐like structures resembling vascular plant forms selected to maximize photosynthetic efficiency (Niklas, 1997). Mosses with diffuse branching around vertical axes resemble vascular plant forms selected to maximize mechanical stability (Niklas, 1997). Regularly or distally branching moss forms resemble vascular plant forms selected to optimize multiple factors including mechanical stability, light interception, water use efficiency and reproductive success (Niklas, 1997). The branching morphotypes of pleurocarpous mosses with two module classes (Fig. 5i,k,n) were nonadaptive in models of early vascular plant evolution (Niklas, 1997) and may have evolved in response to constraints imposed by newly available ecological niches during the rise of flowering plant‐dominated ecosystems (Newton, 2007). For instance, such architectures evolved repeatedly in association with epiphytic substrates, whereas other branching forms (e.g. Fig. 5m) are strongly associated with terrestrial substrates (Bell *et al*., 2012). Together, the data suggest that both developmental innovations and environmental drivers contributed to branching form diversification in mosses, and that mosses and vascular plants are likely to have diversified in response to distinct selective pressures.

### Mechanisms for branching form diversification

The genetic mechanisms regulating apical cell identity in mosses are not yet known, but in *Physcomitrella patens*, strigolactone synthesis at the base of gametophores suppresses branch initiation (Coudert *et al*., 2015), and we speculate that the shift from terminal to basal module repetition (Fig. 5, innovation 1) may reflect a loss of activity. Likewise the developmental and genetic mechanisms regulating the reproductive transition in mosses are unknown, but temperature and day length are inductive cues (Nakosteen & Hughes, 1978; Chopra & Bhatla, 1983) and the switch to pleurocarpy may have involved overriding such cues in primary modules (Johnson *et al*., 2016). Changes in branch distribution patterns can be driven by perturbations in auxin, cytokinin and strigolactone concentrations and by perturbing callose biosynthesis (Coudert *et al*., 2015). The results presented here provide an explicit framework within which to identify the contribution of such pathways to the diversification of branching forms, and the mechanisms underlying convergent evolution in plants.

## Author contributions

Y.C, N.E.B. and C.J.H. designed the study; Y.C. carried out morphological character analysis and scoring and phylogenetic mapping; N.E.B. carried out phylogenetic and ancestral character state reconstruction analyses; Y.C., N.E.B., C.E. and C.J.H. undertook data analysis and interpretation; Y.C. and C.J.H. wrote the manuscript with help from N.E.B; and C.J.H. supervised the study.

## Supporting information

Please note: Wiley Blackwell are not responsible for the content or functionality of any Supporting Information supplied by the authors. Any queries (other than missing material) should be directed to the *New Phytologist* Central Office.

**Fig. S1 **Diagrams illustrate the diversity in modularity, branch distribution patterns, module repetition and perichaetium/sporophyte position.
**Fig. S2** Most likely tree showing relationships between 175 species of Bryopsida.
**Fig. S3** Bayesian majority consensus tree showing relationships between 175 species of Bryopsida.
**Fig. S4** Simplified phylogenetic tree showing directionality in character state transitions.
**Fig. S5** Distribution of species between morphotypes.

**Table S1** List of species studied including authorship, order, GenBank numbers and vouchers of analysed herbarium specimens

**Table S2** Bayesian reconstruction of ancestral states for characters II, III, IV and V at selected nodes

**Table S3** Architectural diversity illustrated in Fig. S1

**Notes S1** Bayesian alignment of nucleotide sequences used to compute Bayesian phylogenetic trees.
