# Supplementary material for: Multiple innovations underpinned branching form diversification in mosses
Source: New Phytol. 2017 May 4;215(2):840–50. doi: 10.1111/nph.14553 (PMC5697605; doi:10.1111/nph.14553)
Supplement: Supplementary file 1 — Fig. S1 Diagrams illustrate the diversity in modularity, branch distribution patterns, module repetition and perichaetium/sporophyte position. Fig. S2 Most likely tree showing relationships between 175 species of Bryopsida. Fig. S3 Bayesian majority consensus tree showing relationships between 175 species of Bryopsida. Fig. S4 Simplified phylogenetic tree showing directionality in character state transitions. Fig. S5 Distribution of species between morphotypes. [file NPH-215-840-s001.pdf]

***New Phytologist* Supporting Information**

Article title: Multiple innovations underpinned branching form diversification in mosses
Authors: Yoan Coudert, Neil E. Bell, Claude Edelin and C. Jill Harrison
Article acceptance date: 27 February 2017

The following Supporting Information is available for this article:

Fig. S1: Diagrams illustrate the diversity in modularity, branch distribution patterns, module repetition and perichaetia/sporophyte position.

Fig. S2: Most likely tree showing relationships between 175 species of Bryopsida.

Fig. S3: Bayesian majority consensus tree showing relationships between 175 species of Bryopsida.

Fig. S4: Simplified phylogenetic tree showing directionality in character state transitions

Fig. S5: Distribution of species between morphotypes.

Table S1: List of species studied including authorship, order, GenBank numbers and vouchers of analysed herbarium specimens. Most specimens were available at the Cryptogamic Herbarium of the Natural History Museum London, others were obtained from the Herbarium of the Royal Botanic Garden, Edinburgh (see separate file).

Table S2: Bayesian reconstruction of ancestral states for characters II, III, IV and V at selected nodes. Average scores (Av.) represent mean percentage probabilities of ancestral character states from three independent analyses. Nodes X, Y, Z have intermediate positions between nodes 1 and 2, and reconstructions show that the shift in module repetition occurred between node Y (the Bryidae, unresolved in the Bayesian consensus) and node 2. Node Z represents the most likely resolution of node Y (with the Bryales sister to the clade represented by node 2; see Fig. S2), and under this hypothesis the shift can more specifically be said to have occurred between node Z and node 2 (see separate file).

Table S3: Architectural diversity illustrated in Figure S1 (see separate file).

Notes S1: Bayesian alignment of nucleotide sequences used to compute Bayesian phylogenetic trees (see separate file).

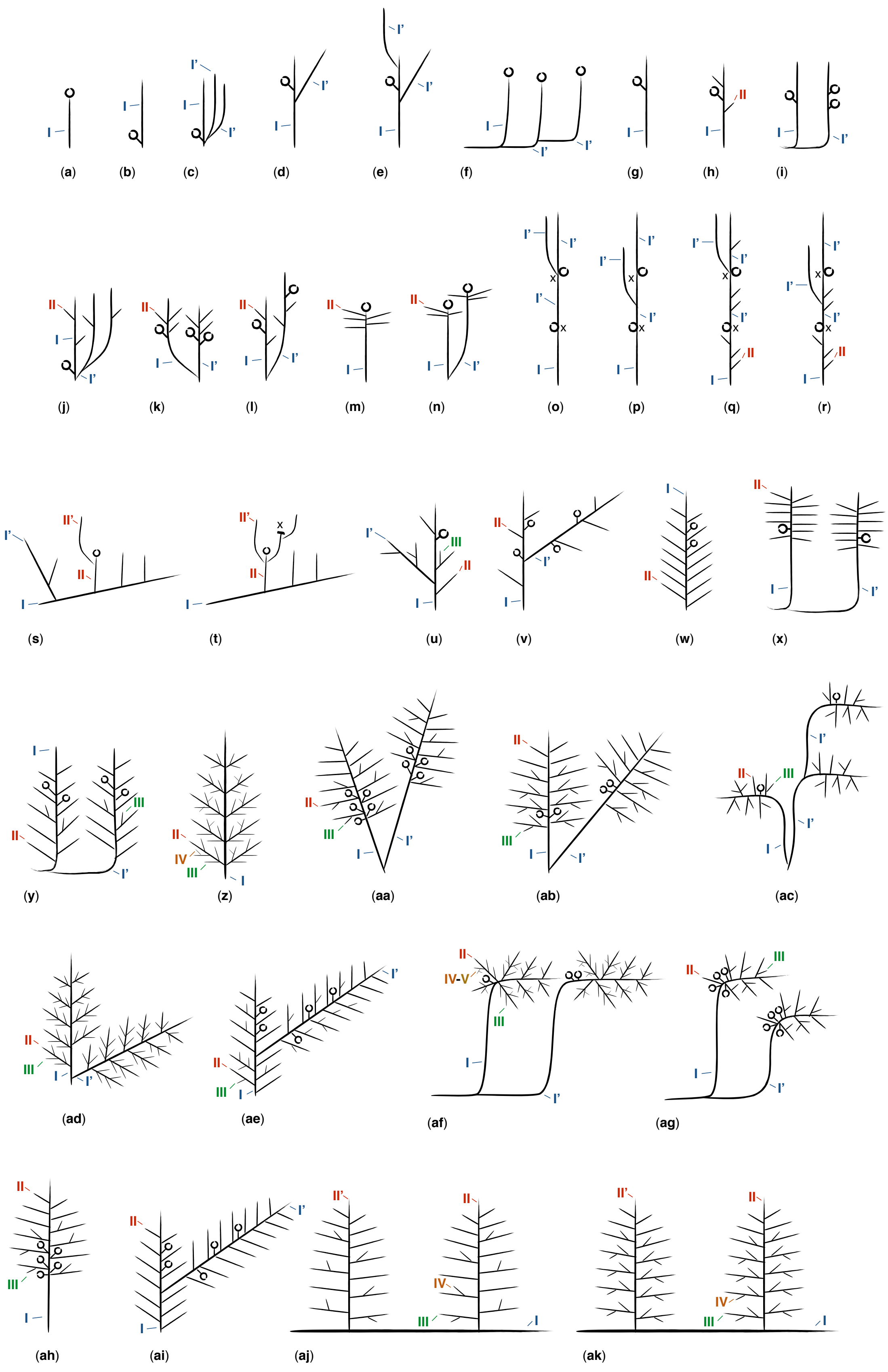

(a)
(b)
(c)
(d)
(e)
(f)
(g)
(h)
(i)
(j)
(k)
(l)
(m)
(n)
(o)
(p)
(q)
(r)
(s)
(t)
(u)
(v)
(w)
(x)
(y)
(z)
(aa)
(ab)
(ac)
(ad)
(ae)
(af)
(ag)
(ah)
(ai)
(aj)
(ak)
I
I'
II
II'
III
IV
IV-V

Fig. S1: Diagrams illustrate the diversity in modularity, branch distribution patterns, module repetition and perichaetia/sporophyte position. Coloured numbers indicate module classes, apostrophes indicate repeated modules, open circles represent perichaetia/sporophytes and crosses indicate a point of growth arrest. Species were allotted to an overall architecture as listed in **Table S3**.

Notes:

(o-r) In these plants, reproductive structures develop at the extremity of primary modules, and primary modules repeat terminally (marked by a "x"). Terminal repetition of primary modules may displace reproductive structures to the side of vegetative modules, which makes them look lateral (as represented on the diagrams) although they are truly terminal. This phenomenon has been well described in Hedwigia ciliata (De Luna, 1990).

(o, q) In these plants, several primary modules may repeat at the extremity of a terminated module. This is represented at the top of the diagrams.

(t) Little hash beneath the cross indicates accidental termination of growth. This phenomenon may induce module repetition as shown on the diagram

Reference:

De Luna E. 1990. Developmental evidence of acrocarpy in *Hedwigia ciliata* (Musci: Hedwigiaceae). *Tropical Bryology* **2**: 51- 58.

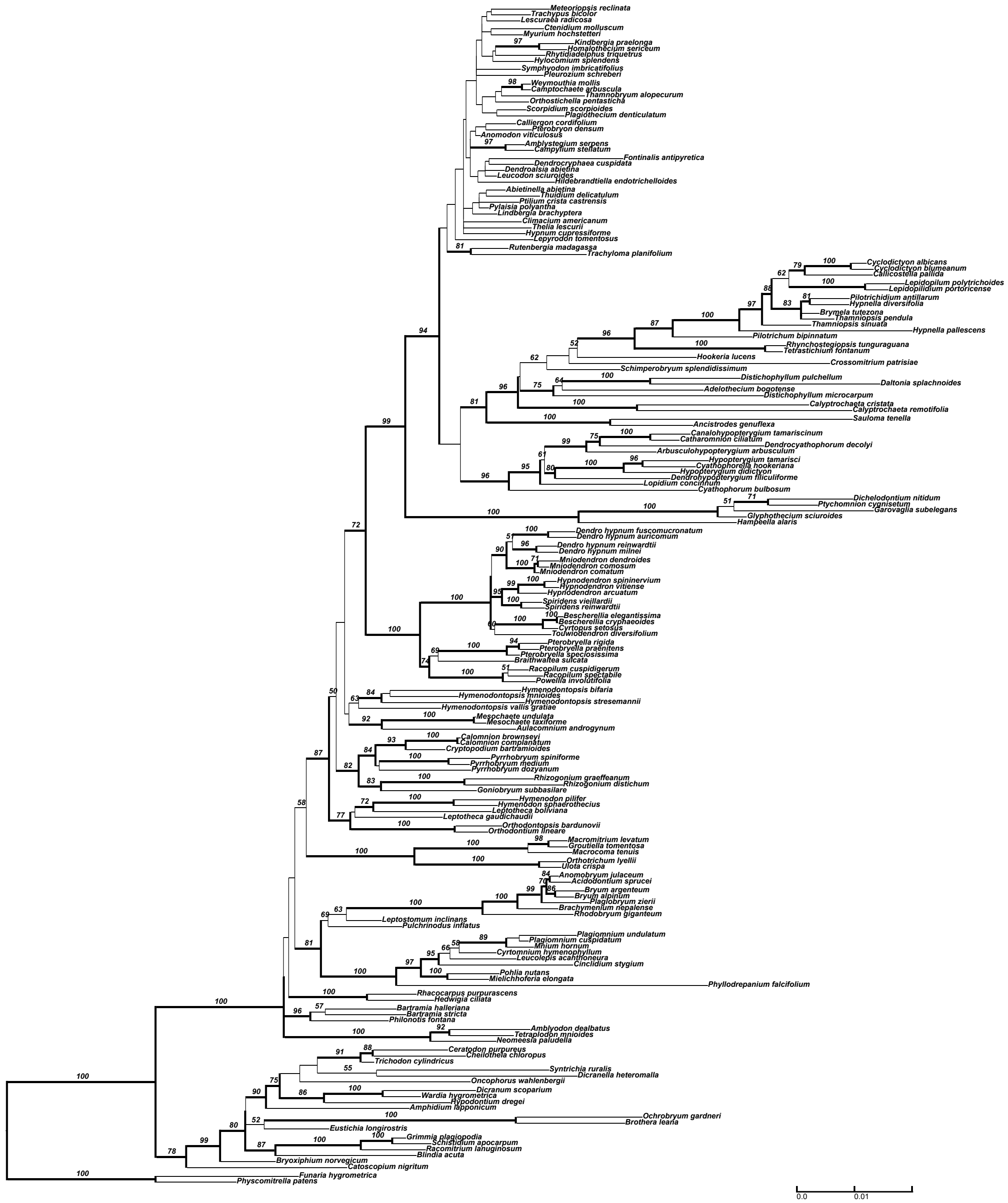

Fig. S2: Most likely tree showing relationships between 175 species of Bryopsida. Phylogenetic analysis was done with *rps4*, *rbcL* and *nad5* sequences using a heterogenous GTR + G model of nucleotide substitution. Numbers on branches are likelihood bootstrap values > 50%. Branches in bold are supported by bootstrap values > 70%. *Physcomitrella patens* and *Funaria hygrometrica* sequences were used to root the tree. Scale bar represents number of nucleotide substitutions per site.

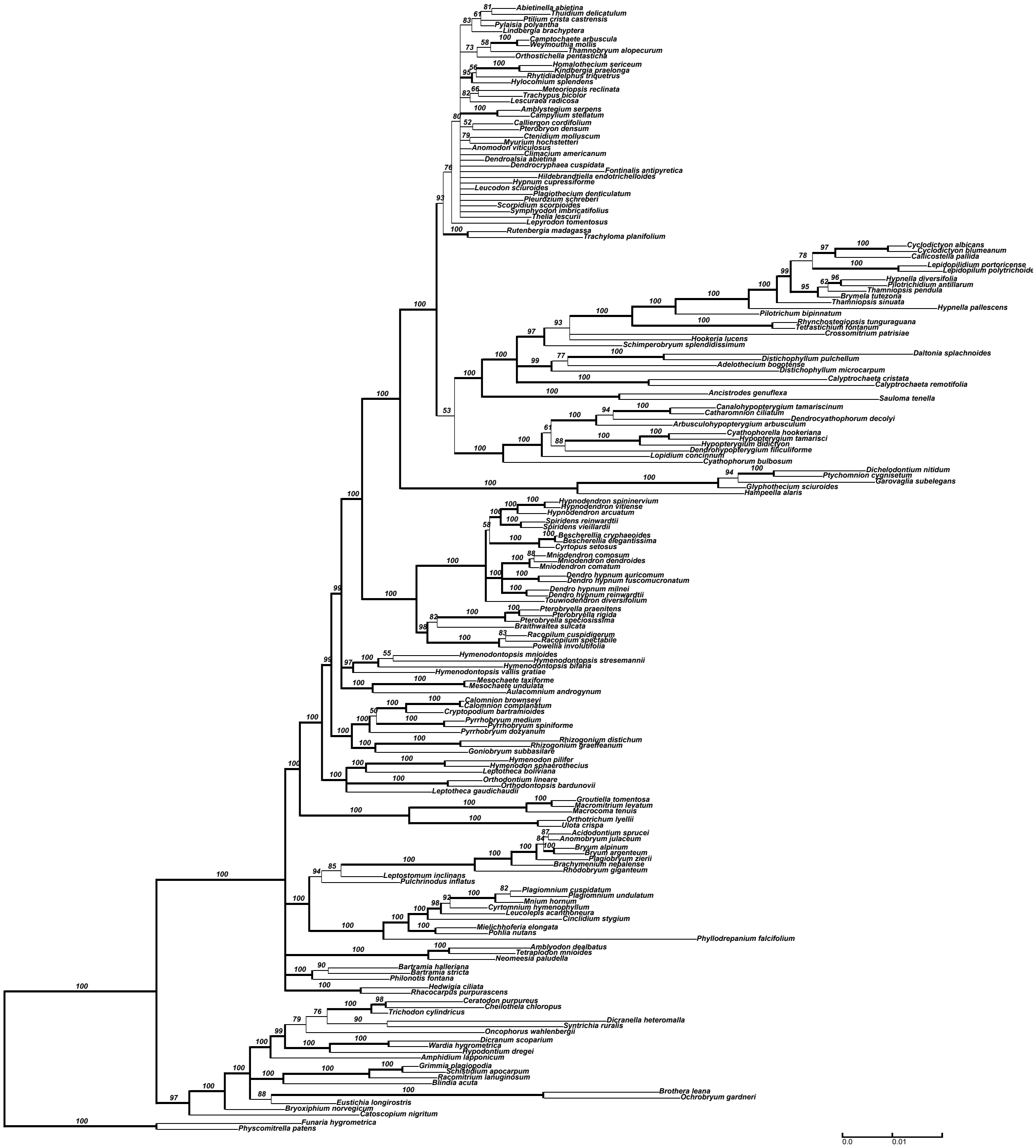

Fig. S3: Bayesian majority consensus tree showing relationships between 175 species of Bryopsida. Phylogenetic analysis was done with *rps4*, *rbcL* and *nad5* sequences using a heterogenous GTR + G model of nucleotide substitution. Numbers on branches are posterior probabilities > 50%. Branches in bold are supported by posterior probabilities > 95%. *Physcomitrella patens* and *Funaria hygrometrica* sequences were used to root the tree. Scale bar represents number of nucleotide substitutions per site.

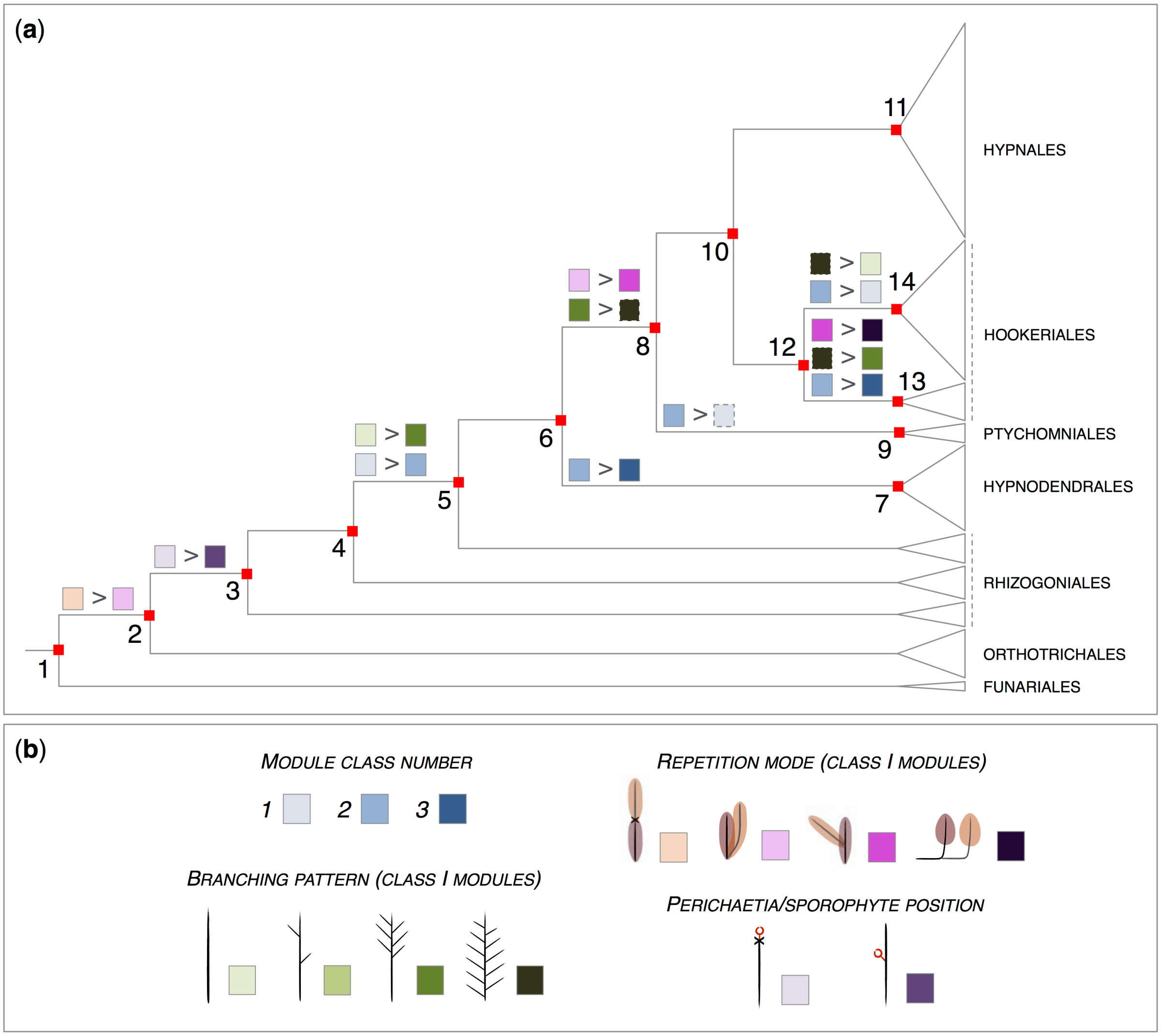

Fig. S4: **Simplified phylogenetic tree showing directionality in character state transitions**. (a) Red squares and numbers indicate critical phylogenetic nodes analysed as in Fig. 4. State transitions with 50% support or more in the ancestral or derived state were represented (as indicated in Table S2), and uncertainty (less than 50% support) is indicated by a dashed box. (b) Key to character states. The number of module classes ranges from 1 to 3. Primary (class I) modules may be unbranched or have diffuse, distal or regular branching patterns. Module repetition modes vary as identified in Figure 2. Perichaetium/sporophyte position may be acrocarpous (terminal) or pleurocarpous (on short lateral branches).

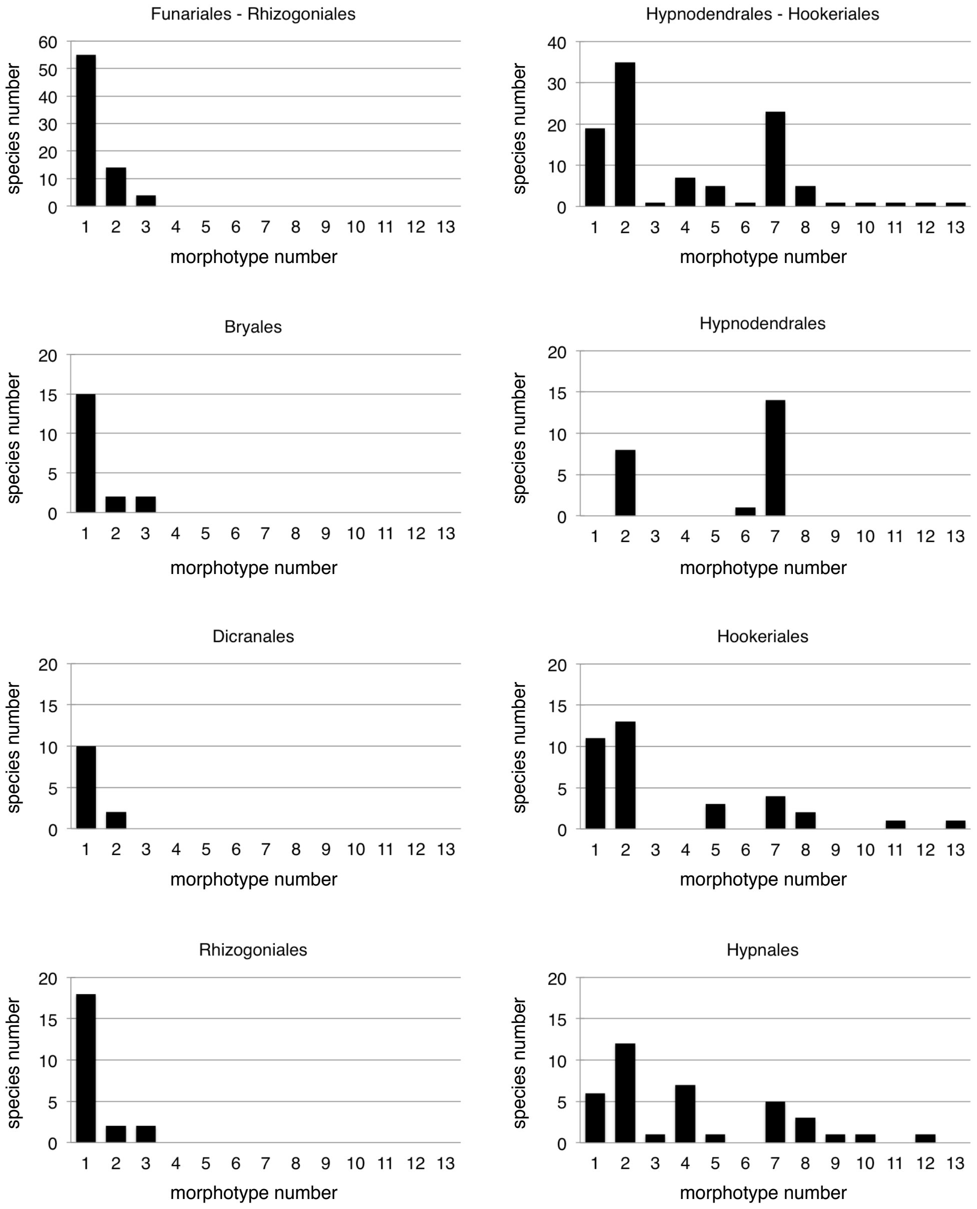

Fig. S5: The distribution of branching architectures is conserved between acrocarpous mosses and early diverging pleurocarpous lineages (Funariales-Rhizogoniales: left half of page) but varies amongst recently derived pleurocarpous lineages (Hypnodendrales-Hookeriales: right half of page).
